# Dr. Ferran’s Experiments in Cholera Inoculation

**Published:** 1885-08

**Authors:** 


### DR. FERRAN'S EXPERIMENTS IN COLERA INOCULATION.

DR. BENJAMIN EDSON, in the *New York Record* says: "It is but natural that the public should take a deep interest in the result of the experiments of Dr. Ferran in vaccinating with attenuated cholera virus. Any prophylactic against a disease so dreaded and feared as cholera, will be hailed as a boon to mankind.

"The progress of Dr.F's experiments is noted most flatteringly by the public press, even Medical Journals echo favorable reports in a half hopeful manner. I fear, however, that those longing expectations are only born of hope. It is true that we vaccinate as a preventive in variola, but we have this fundamental principle to start with, that one attack of variola gives, as a rule, exemption against any subsequent attack. If one attack of cholera conferred exemption against a subsequent attack, we could look to protective vaccination with much more confidence. From what I have seen of the disease, and from those who have observed all the epidemics of the disease that have occurred in this country, I find no warrant for belief that one attack of cholera may not be followed by another in the same person. Most authors make no reference to this point. Goodeve, in "Reynolds' System of Medicine," vol. 1, p. 392, states that there are numerous cases on record, of persons who have had cholera more than once.

"Roberts, (part 1, p. 219) says: "One attack does not afford protection against another." So far as I am aware, this is the uniform testimony of all who have expressed their views on this feature of cholera.

"Now, if an unmitigated attack of Asiatic cholera in its worst form confers no exemption from a subsequent attack, I see no reason to hope that vaccination with attenuated virus will confer any possible degree of exemption or protection."

In Alcira, out of a population of 20,000, Dr. Ferran inoculated 9,100, and reinoculated 7,500. There was no distinction as to classes, rich and poor submitting alike to the operation. Out of 320 cases of cholera reported, up to the date this was telegraphed to the *Herald*, 130 died, and 138 recovered, and 52 were under treatment. One hundred and twenty, out of the 130 deaths occurred amongst those who had not been inocculated. Seven deaths occurred in those who had been inoculated, and three deaths occurred in the reinoculated class. That certainly looks like a very large measure of "protection." At any rate, under similar circumstances, we would "inoculate." The *New York Medical Record* says on this point, "Assuming these figures to be correct, approximately, we may take the smaller percentage of cases and deaths among the inoculated to represent the prophylactic effects *of a feeling of security* caused by the operation. In other words, inoculation may be interpreted as a kind of *faith preventive.* A factor of importance, also, is probably the inoculation of the more intelligent and careful. Altogether, we can see, as yet, nothing in the least demonstrative of the results of Ferran's inoculation."

